# Identity in Personal Recovery for Mothers With a Mental Illness

**DOI:** 10.3389/fpsyt.2019.00089

**Published:** 2019-03-08

**Authors:** Rochelle Helena Hine, Darryl John Maybery, Melinda Jane Goodyear

**Affiliations:** ^1^Mental Health Services, South West Healthcare, Warrnambool, VIC, Australia; ^2^Department of Rural Health, Monash University, Moe, VIC, Australia

**Keywords:** gender, identity, mental illness, motherhood, personal recovery

## Abstract

Developing a “positive identity” is considered a core component of personal recovery, and mothering offers meaning in life and a valued identity. Few studies have highlighted the factors influencing identity within a personal recovery paradigm for mothers with mental illness. This study explores how mothers describe their identity in relation to recovery, including the factors that influence identity. Using constructivist grounded theory methodology, in-depth interviews were conducted with 17 women who were mothers and experienced mental illness. Women defined their self-concept broadly, accentuating motherhood, but also including vocational, community and social roles. Analysis revealed six categories: defining self, becoming a mother, being a “good” mum, feeling different, doing it my way and speaking out. Valuing identity in parenting was found to be linked to recovery. Services may facilitate personal recovery by supporting mothers to enhance a self-concept associated with mothering, as well as other diverse attributes and roles.

## Introduction

Personal recovery is a unique journey for those experiencing a mental illness, however common underlying characteristics and processes are now becoming better understood (1). Distinct from clinical recovery, which emphasizes a remission of psychiatric symptoms, personal recovery is grounded in the subjectivity of people who have live experience of mental illness (2). Personal recovery is concerned with holistic life functioning and social participation and agency, regardless of the presence of ongoing symptoms (3). Recovery experiences are unique and individual, and also differ for men and women due to the influence of gender (4).

Identity, and particularly the development of a positive sense of self that is less illness dominated and more strength based, is one of the key processes of the CHIME personal recovery framework developed by Leamy et al. (1). Resulting from a systematic review of personal recovery literature, the CHIME framework highlights Connectedness, Hope, Identity, Meaning and Empowerment as core categories that transform in a recovery journey. The significance of redefining and reclaiming a valued identity may comprise “determining the direction of one's life, grieving for lost opportunities, and yearning for belonging and acceptance” (5).

The ways in which mothers with mental illness describe their identity and the influences upon it within the process of personal recovery, has remained peripheral within recovery discourse. The aim of this study is to explore the ways in which mothers with mental illness describe their identity, and the factors that support or hinder development of a positive identity. Critical research aims to reveal and question social inequalities that are embedded within stereotypes (6). This current study adopts a gendered lens to examine the identity experiences of mothers with mental illness, and to investigate multiple identity possibilities including and beyond motherhood.

### Identity Theory

Identity is broadly defined as one's self concept and is constructed through self-awareness of what and who one is “like” and “not like” (7). Identity is constructed within one's social and cultural context (8) and is therefore related to the construct of connectedness. Identity is a highly contested concept within feminist theory. An overarching theme is that gender based identity is predominantly oppressive. Butler (9) has problematized the concept of gender identity as the reproduction of the subordination of women, and explored the ways in which it is reproduced through linguistics. She also questions whether identity is a stable and continuous entity, and if it is static, the social regulatory structures that produce this outcome:

‘To what extent is ‘identity’ a normative ideal rather than a descriptive feature of experience? And how do the regulatory practices that govern gender also govern culturally intelligible notions of identity? In other words, the ‘coherence’ and ‘continuity’ of ‘the person’ are not logical or analytic features of personhood, but rather socially instituted and maintained norms of intelligibility' (p.23).

Côté, defines identity as multifaceted and states that “manifestations of identity exist at three levels of analysis; the subjectivity of the individual, behavior patterns specific to the person and the individual's membership in societal groups” [(10), p. 8]. Within psychology, these differentiations have more broadly been separated into two categories: personal and social identity.

Personal identity relates to the internal characteristics including attributes and values that one recognizes as inherent to one's self concept. Social identity meanwhile, reflects how individuals view themselves as affiliating with and belonging to particular societal groups. Vocational, family, community, political, or gender-based categorizations are realms in which social identity may exist.

A fundamental debate in identity theory is whether the self is essentially a stable and enduring entity, or whether it is constantly changing and evolving (7). According to self-schema theory, the core identity constructs of an individual are thought to remain stable over time, although there may be some change in the more peripheral aspects of one's identity (11). Rosenfield (12) used schemas relating to self-salience, to explain disparities in mental health outcomes across gender, race and class, where a social determinants framework was ineffective in accounting for results that were inconsistent with structural inequality. Self-salience is associated with the relative importance individuals place on self, vs. the collective (13, 14). Rosenfield (12) cites the gendered socialization processes that contribute to the social construction of femininity and masculinity, and recognizes how these differ in relation to race and culture (15). Historical and enduring social conditioning underlies the gendered internal self-salience tendencies that predispose white women to experiencing more internalizing problems (e.g., depression) and white men to have a greater prevalence of externalizing problems (e.g., aggression) (12).

In contrast, self-categorization theory is based on the assumption that individuals' identity can and does change and evolve in response to the social and environmental context in which people live, and in response to external and internal processes associated with major life events. Ontorato and Turner (7) compared the two theories and found evidence to support self-categorization theory, with two studies demonstrating “the dynamic nature of self” (p. 276) that is context dependent. Self-categorization is a useful theory to inform exploration of the influences on identity development occurring through recovery from mental illness. Underlying assumptions imply that recovery emerges through social and psychological processes of personal change and development (1). The depth and breadth of self-reflection and change embedded in many of the subjective narratives of those with lived experience in mental illness and recovery, suggest that recovery entails a transformation at a deep psychological level, not merely some pruning or enhancement on the margins. Thus, self-categorization theory relates to this study.

Although gender identity can be a source of solidarity amongst women, it is more frequently problematized within feminist debate as a site for the reproduction of existing power differentials and maintenance of patriarchy. Within feminist theory:

“…the reproduction of normative identities cannot be understood simply as a question of positioning within language but as a lived social relation that necessarily involves the negotiation of conflict and tension” [(16), p. 185].

For women who are mothers with mental illness, those conflicts and tensions may be the precipitant as well as the result of the psychiatric diagnosis and subsequent treatment and recovery. One method of developing deeper understanding of lived social relations is through amplification of the voices of those women who are marginalized in dominant discourse. This study aims to contribute to that endeavor.

### Mental Illness and Identity

Mental illness has been characterized as a loss of self (5), as psychiatric symptoms may conceal or distort an individual's skills, knowledge, values and attributes. Social identity can also be disrupted as the illness often manifests in ways that prevent people from continuing in social roles that they had previously occupied and enjoyed (17). Individuals have spoken of enduring confusion, grief and regret as they seek to reconstruct a sense of self and reconcile the differences in their identities before, during and after the mental illness experience (18).

Researchers have explored the relationship between psychiatric symptoms and disruptions or incongruence in self-concept (19, 20) including models of conceptualizing and mitigating the impact of complex trauma on ones' identity and healing (21). Wisdom et al. (5) found loss of self to be the most prominent focus of narratives, with the illness “often described as taking away…their previously held identity” (p. 491).

Feminist writers have observed that historically, society's response to mental illness in women has resulted in sanctions for deviance and non-conformity to the prevailing cultural expectations [e.g., (23)]. A focus on social “integration” and adaptation of the individual, rather than advocacy for social change to create more equal and accessible social environments that foster diversity and inclusion, are evident in critiques of the recovery paradigm (4, 22).

### Mothering With a Mental Illness

Mothering with mental illness is increasingly common (24, 25). Studies conducted with mothers with mental illness have emphasized the importance of a mothering identity to women in providing meaning and purpose (26), love and connection (26), and fulfillment (27).

Researchers have highlighted that parenting stress can compromise mental health (28–30), and discovered that mothering confidence and competence can be undermined by the scrutiny and prejudice imposed by over-zealous service providers (26, 31) and family members (32), who are often operating within a risk aversion framework.

Shor and Moreh-Kremer (33) emphasized the strengths for women with mental illness in being able to claim a normative maternal identity, thereby reducing their vulnerability to stigma and alienation. Within that study mothering identity was compared to mental illness identity, without consideration of other potential sources of identity in women's lives. While there may be individual benefits to conforming to gendered norms, if this is through developing a public persona that is incongruent with one's internal value system, and acceptance of oppressed social status, there will also be psychological costs (23).

## Aim

The aim of this study was to explore how a cohort of Australian women who were mothers with mental illness described their identity, and how this related to personal recovery from mental illness. The research furthermore aimed to explore the factors and processes that mothers with mental illness describe as influencing their identity.

## Method

Constructivist grounded theory (CGT) methods were employed to gather and analyse rich descriptive qualitative data (34). Grounded in feminist understandings of gender inequality that can result in and exacerbate the effects of mental illness, the research aimed to amplify the voices of women from a marginalized population group (35). Congruent with feminist approaches to research, CGT challenges the objectivity claims of positivist methods, instead advocating for transparency in acknowledgment of the values, perspectives, experiences and biases of the researcher which all influence the research decisions, processes, and outcomes (34). CGT emerged in the 1990's [e.g., (36, 37)] in response to the epistemological assumption that if “social reality is multiple, processual, and constructed, then we must take the researcher's position, privilege, perspective and interactions into account as an inherent part of the research reality” [(34), p. 13]. While traditional grounded theory methods emphasize that researchers arrive at their own data fresh, without prejudice or influence from previous studies (38) this sequencing is not required for undertaking constructivist grounded theory as the background research and theoretical context investigated, contributes to the unique subjectivity of the researcher. As all experiences in the social world influence the perspectives, language, assumptions and biases a researcher brings to each project (34), it would be exceedingly ineffectual to refrain from reviewing literature as just one of infinite potential influences. Hence in this study, literature on identity and gender were explored prior to the data collection with participants, with a more focused search ensuing after the categories were formulated.

### Procedures

Following ethics approval from two health service and one university Human Research and Ethics Committees, mental health clinicians from a regional clinical mental health service supported recruitment processes by promoting the study amongst eligible women on their caseloads. Steps were taken to ensure no harm or distress was caused, and that women felt comfortable to end or pause the interview at any time. All interviews were conducted by a researcher who was also an experienced social worker employed as a senior mental health clinician and informed consent was obtained in writing. Local services guides were developed and distributed to participants in the event that the interviews triggered psychological response requiring follow up.

Nine women were recruited via clinical mental health services, and the remaining 8 from the general community via social and print media (promoted on Twitter and in local newspaper articles). Interviews were conducted by one researcher (RH), and consistent with CTG procedures, four of these 17 women were interviewed on more than one occasion as part of theoretical sampling. These participants were invited to participate in a second interview due to specific characteristics they possessed, that meant they were able to provide data that could increase understanding of phenomena relating to emerging codes. A total of 21 interviews were conducted, the mean duration being 42 min. The participants were at different stages of recovery. None of the women were in an acute crisis or experiencing active symptoms at the time of the data collection, although one woman had been hospitalized within the last month, and 6 were engaged with a clinical mental health service at the time of the interview. Three women were supported by a community mental health service and the remaining eight reported not receiving any specific mental health service at that time.

Interviews were conducted in person within a confidential space at various community health venues that were accessible to participants. Interviews were audio recorded and transcribed verbatim. The interview schedule was open ended and flexible, containing questions pertaining to women's experiences of personal and social identity. The interview schedule is available as a Supplementary Material. To set the context, women were asked about their mental illness and recovery journeys. Women were then asked broad questions regarding how they describe themselves, their social roles, personal characteristics, if and how the way they see themselves has changed over time, about their mothering and parenting, how they envisage others see them, and what or who influences how they view themselves in various settings (e.g., work, mothering/family, community). The total recruitment and interview time frame was from July 2015 to February 2016.

### Data Analysis

Data analysis occurred concurrently with data collection, coding and reflection beginning immediately after transcription of the first interview and continuing after each interview. Analysis consisted of initial coding following close reading of all data on multiple occasions. Memo writing, purposive sampling, focused coding, mind-mapping were subsequently undertaken to enable development of categories (34), hence the categories emerged directly from the data. Regular dynamic discussion amongst the three member research team stimulated analysis and questions relating to the emerging categories. This led to second interviews with some participants to provide additional details to define the properties of emerging categories, as consistent with purposive sampling.

### Participants

Participant characteristics are summarized in Table 1. A total of 21 interviews were conducted with 17 women who were all mothers with a psychiatric diagnosis. The women were a heterogeneous group and varied in relation to their living arrangements, socio-economic status and cultural background. Ages ranged from 23 to 53 year, with an average age of 36.29.

**Table 1:** Participant characteristics.

**Age; cultural background**	**No. of children and age**	**Housing type and lives with**	**Self-reported diagnosis and did diagnosis occur prior to motherhood?**	**Income source**	**Current mental health service engagement**
38; Irish and French	3:10 years, 8 years, 6 years	Private rental Lives alone	Bipolar, OCD, BPD Yes	Government payment	None
35; Australian	2:2 years, 10 months, pregnant at time of interview	Own home on farm Lives with husband and children	Anxiety and depression No	Farm income	None
44; Australian	2:13 years, 11 years	Own home Lives with husband and children	Anxiety and depression No	Husband's full time wage	None
39; English and Australian	1:15 years	Own home Lives with partner and child	PND, anxiety and depression No	Own full time wages	None
39; Finnish and Italian, adoptive family Australian	1:14 years	Private rental Lives with child	BPD and bipolar disorder. No	Government payment and part time wages	Community MHS
40; Australian	1:2 years	Public housing Lives with child	Drug induced psychosis Yes	Government payment	Clinical MHS
29, Aboriginal	8:15 11 10 8 4 3 plus 2 younger in foster care	Public housing Lives with eldest 4 children	Anxiety and depression, paranoia, bi-polar disorder No	Government payment	Clinical MHS
31; Aboriginal	6:14 years, 12 years, 9 years, 4 years 2 years, 8 months	Transitional housing Lives with youngest 3 children	Anxiety, PTSD No	Government payment	Clinical MHS
43; Australian	1:15 years	Own home Lives with husband and child	Bipolar Disorder Yes	Government payment	Community MHS
26; Australian	1:6 months	Private rental Lives with partner and child	Bipolar Disorder Yes	Partner's full time wage	Clinical MHS
34; Australian	3:9 years, 8 years, 5 years	Private rental Lives with children	PTSD and depression, PND No	Government payment	None
53; Australian	3:17 years, 16 years, 13 years	House on the family owned farm Lives with husband and children	PND and depression No	Farm income, and wages from 3 part time jobs	None
28; Australian	2:5 years, 2 years	House on the family owned farm Lives with husband, children and boarder	Depression Yes	Part time wages and farm income	None
44; Australian and English	1:9 years	Private rental Lives with mother and child	Depression and anxiety No	Government payment	Community MHS
23; Australian	1:10 months	Own home Lives with partner, step child and son	Depression and anxiety Yes	Full time wages and partners' wages	Clinical MHS
45; Australian and German	2:14 years, 12 years	Own home Lives with husband and children	Anxiety, OCD and depression Yes	Part time wages and husband's full time wage	None
26; Caucasian	1:14 months	Parents' home Living with parents and child	Depression, Anxiety and PND No	Husband's full time wage	Clinical MHS

*BPD, Borderline personality disorder; MHS, Mental Health Service; OCD, Obsessive compulsive disorder; PND, Postnatal depression; PTSD, Post traumatic stress disorder*.

## Results

The study resulted in identification of six categories surrounding the concept of identity. The categories name psychological or social processes that were predominant within the data collectively. “Defining self” explores how women describe their self-concept and highlights the important elements of personal and social identity. As mothering was a core component of participants' identity, the categories “becoming a Mother” and “being a ‘good Mum’” illuminate the relationship between women's mothering role, their mental illness and their recovery journey. The relationship between an illness identity and a mothering identity is explored in these sections. The category “feeling different” focuses on the women's experience of lacking a social identity and the implications of this on their personal identity. The final two categories reflect women's recovery progress, and highlight the importance of developing a positive identity, for attaining other personal recovery outcomes such as empowerment and meaning in life.

### Defining Self

In describing their sense of self, all of the participants sought to contextualize their current temporal personal identity within their life histories, and inter-relational experiences, beginning in childhood. Ten women shared accounts of trauma from interpersonal violence occurring within childhood and/or adult relationships, and it is not known whether the remaining women may have also experienced violence but not disclosed. Through articulating key formative events, the women sought to construct identity narratives that provided meaning to explain their current circumstances, including their mental illness diagnosis.

Personal and social identities were described by participants as changing and evolving, while also containing stable components that persisted over time as illustrated by the assertion “I've always been this way” (P7). Participants described their identity in relation to their personal attributes, social roles and key relationships. Not all women were able to eloquently describe themselves, however there were exceptions:

“(I'm) a woman, a mother you know, I'm a feminist, I'm an atheist, I'm left wing, very left wing in a lot of ways. I'm very politically minded, I'm very also socially conscious I guess. I do a lot of social activism. I'm bisexual so I've done a lot of campaigning for marriage equality and stuff like that. I'm very creative… I do a lot of crafty stuff. I love having kids because it gives you an excuse to colour in… I do a lot of puzzles, I watch probably way too much television and movies, I'm a huge film buff. I love politics as well” (P11).

Moral characteristics such as honesty, independence, generosity and creativity were viewed positively in the ways in which women viewed themselves, and they validated this through reflecting on how others might see them “I think people would describe me as nice and caring” (P13). Recognition of their own resilience was also evident (*n* = 9) in comments such as “…sometimes I look back on what I've coped with and I think, wow” (P5).

For other women, there was recognition that attributes that had been characterized as strengths prior to the mental illness experience could also become barriers to the help seeking that may be required to address mental health challenges:

“I would describe myself as very independent. Probably too independent… I like individual sports like running and tennis. I've never really played team sports. I like to achieve, like at work I became fairly obsessed with achieving at work. But that was another thing after having the baby. It was very different…” (P2).

The social roles that the women identified embodying were relational, vocational, and community orientated. They included mother, sister, daughter, partner, friend, worker, health professional, student, mental illness advocate or educator, committee member and volunteer. Relationships with others in these spheres contributed to how they viewed themselves, and their personal identity could be particularly susceptible to messages they received from significant others regarding their performance within those roles.

Four women referred to their religious affiliation as being important in defining their moral and ethical framework. Although three women spoke of attending church related activities, they did not view themselves as sharing many characteristics with other members of their church except for their religious beliefs, therefore this was salient for personal rather than social identity.

Cultural identity was discussed by five women, two of whom were of Aboriginal descent (P7 and P8). Cultural disconnection was apparent with one of the women who indicated she knew little of her heritage as her Aboriginal father had died when she was young. Another Aboriginal woman had experienced significant trauma during childhood and in adult life and had lacked opportunities to develop cultural knowledge or connection, stating “I want to know my culture, but I don't want to *do* my culture” (P8).

Participants articulating devalued personal identities were more likely to be in roles or relationships where they derived little pleasure or fulfillment:

“I have issues with my work and because my boss is bully… when they cut my hours I took it really personally and I couldn't go to work the next day–I just cried” (P16).

Some women had difficulty describing themselves, identifying their strengths and imagining themselves in the future: “at the moment I can't see past tomorrow… I'd really like to enjoy things in my life a little bit more” (P15).

Identity across different domains could manifest in either positive, socially valued ways, or as negative and socially devalued. For example, a mothering identity could be positive if one perceived oneself to be a “good” mother, and felt a sense of belonging to a mothers' group. Alternatively, a mothering identity could be experienced as negative if one considered herself to be deficient and incompetent, thereby judging herself as a “bad” mother. Similarly, if a woman held a position of esteem within the community as a committee member in a sporting club, this would foster positive personal and social identity across other domains, whereas a lack of community recognition or a sense of social distance could contribute to a devalued identity reinforcing isolation and social exclusion.

### Becoming a Mother

“When I gave birth, I felt connected to something bigger and stronger than myself. I'm not religious, but I'm spiritual. So I felt connected to the bigger scheme of things like connected to other women and the feminine force of the universe” (P11).

Becoming a mother was a particularly significant component of women's self-concept. It could transform a woman's personal and social identity, fostering a deep sense of connection and meaning. However, it could also negatively impact a woman's view of herself if she struggled to feel competent in the role. Diversity was apparent in the responses the women had to embodying a mothering role and identity. For some it was an identity they immediately embraced and had always expected. The majority of participants (*n* = 12) had always had aspirations of parenting, and one woman with her partner had been planning the pregnancy for some time, including changing psychiatric medication and exploring fertility options. Having a baby brought a sense of empowerment and fulfillment for 8 of the participants. For one woman it was an opportunity to exercise her own autonomy and make choices that would not necessarily be endorsed by her own mother;

“I breastfed until 21 months and I loved it. And my mum said ‘don't you think it's time you gave it up?’ at six months. And I'm like, hey, I felt something was right” (P5).

However, the experience did not always live up to expectations, especially in the early days and weeks.

“I hid it from the maternal and child health nurse that I wasn't coping and then on the Friday I'd had enough and so I rang (family services worker) and said ‘I need your help’” (P10).

For four women, all of whom had relished the idea of motherhood since childhood, childbirth was accompanied by debilitating postpartum depression when they had thought they were emotionally prepared. This left them with guilt and regret for the aspects of early parenting that they missed.

Experiencing breastfeeding difficulties was a source of significant stress and eroded self-confidence for four women. One woman delayed disclosing her decision to bottle feed to her new parent's group, fearing social rejection:

“It took me probably two weeks to tell them that I wasn't breastfeeding, because I was just so anxious about it because everyone else was breastfeeding and I was like, oh they're going to want to kick me out of the group, yeah they won't want to talk to me ever again. But once I told them they were really supportive” (P17).

Having responsibility for a child prompted two women to re-assess their social behavior and temper their anger. They explained how accepting the responsibility associated with caring for an infant had led them to cease drug use, necessitating the severing of social relationships that would undermine this new healthy lifestyle choice. This assisted in them feeling greater competence in managing emotions, which led to a more positive identity.

Becoming a mother could have positive or negative implications for the women's personal identity. This was partially influenced by the availability of supportive relationships and assistance to adjust to the early parenting phase. How women perceived themselves to be performing in the mothering role profoundly shaped their identity, as did the quality of their relationships with family members and health professionals and the ways in which their inherent value was reflected through interactions within these relationships.

Another factor was whether the women's mental illnesses emerged before or after they became a mother. While 6 women had been given a psychiatric diagnosis prior to becoming a mother, an additional 7 participants expressed the belief that the mental health issues that eventually led to the subsequent diagnosis (after motherhood) had existed for many years, in some cases from childhood or adolescence. In these situations women described the mental illness label as providing an explanation for psychological, cognitive and behavioral events, along with the opportunity for enhanced social identity through developing peer relationships.

### Being a “Good Mum”

A “Good Mum” was defined as accepting responsibility for one's children, prioritizing her children's needs over her own, being present and responsive and “making it fun” (P7); using humor and actively engaging in play. Attending to children's emotional needs and being available was a part of this: “I want to be the person that my kids will come and talk to me when something's going on, you know. Instead of hiding that away” (P7). All of the women interviewed expressed a desire to be identified by others as a “good mother.” Furthermore, they wanted to embrace this label for themselves, although for three participants reconciling their thoughts and emotions regarding the mothering role with an ideal self-as-mother, was problematic. One woman rationalized that she wasn't a good mother because

“…most of the time I feel like I'm just getting through… it's the extras that play with the mind and question how well you are doing. Am I stimulating them enough? Am I doing the right things for learning at this age?” (P2).

Characteristics of being a “good” mother ranged from meeting children's basic needs for nutrition, sleep and intellectual stimulation to being physically and emotionally available to children; “a good mother is showing love to their child, their little one, talking to them, validating their feelings… understanding his point of view” (P5). It involved persisting through hard days. Persisting entailed getting up in the mornings despite exhaustion or symptoms of depression, putting “a smile on a lot in front of them and for them” (P7) and for two mothers, remaining in undesirable employment to provide financial stability. In speaking about striving to be a good mother, two participants referred overtly to the sexism embedded in their own interpersonal relationships that saw them taking primary responsibility for child rearing while their husbands enjoyed more leisure time.

Eight women expressed sadness and regret regarding their own upbringing. Attributing their subsequent mental illness to the cumulative impact of trauma, abuse, neglect and disadvantage, participants felt that if they had been raised in a family with a responsive adult figure attentive to their needs, their lives may have taken a different path. This fuelled a desire to be present and considerate and sensitive to their children's traits, strengths and needs, even within challenging socio-economic circumstances.

“Making sure I do a good job and (my child) gets a good education and just the little things, speaking to him nicely. I've never ever yelled at him, I don't believe in that and I don't like people who smack their children” (P6).

Despite best intentions, there were times when women's stress levels were high and their mental health was compromised. They became aware of how difficult this high expectation of parenting was in such times. For five women, serious physical health conditions such as pneumonia also took a toll. At this time children.

“…learnt to become very independent and it was hard to parent them at that time. I didn't have the energy to discipline them and I noticed a lot of things went out the window—just their manners and the way they behaved and stuff—like I just couldn't be a good parent” (P16).

Breaking the pattern of cycles of intergenerational poverty, trauma and substance use were important to women in demonstrating their parenting attributes. They hoped for easier life circumstances for their children and hoped to guide them toward healthy choices:

“It's being able to see beyond their pain and just walk with them and be their friend and guide them and show them this is what Mum and Dad have been through and this is why we don't want you to go down this path” (P1).

Viewing oneself as a good mother therefore had a positive impact on personal identity, while feeling incompetent or guilty had the opposite effect. Women used different measures to assess their parenting capabilities however an increased capacity for self-reflection and self-compassion was associated with greater progress toward recovery.

### Feeling “Different”

A sense of disconnection and alienation from peers and family members pervaded development of a valued and intact self-concept for a number of women and they related experiences stemming from childhood to illustrate this. For some this was characterized by additional sensitivity, “I was a very clingy needy child” (P16) or having different needs and abilities to siblings “when we were younger I would clean (my sister's) room or do stuff for her just so I could spend time with her. Because we're very different” (P10). These examples suggest an unmet need for nurturing and connection in childhood. Resulting from this was a fragmented sense of self that was dominated by rejection.

For others it was about possessing a unique skill set or perspective on life, “the way my mind works I've never known anything different…and because of that I got treated different” (P7). Being able to deflect childhood labels of deviance and learning to embrace their own uniqueness fostered a sense of wellbeing, but was difficult to achieve outside the context of a supportive relationship. Women who felt validated by supportive intimate partners were better able to reflect of their childhood experiences of exclusion and externalize the cause of this experience.

### Doing It My Way

This category was associated with increased confidence in one's experience and competence in mothering and signified a recovery milestone. Over time, women's self-awareness grew, and they became more insightful as to their own strengths, values and needs. This informed the resources and strategies they accessed to support their mental health and wellbeing. They gradually became proficient at seeking the support they required, whether that involved psychiatric medication, talking therapy, social connection, creating art, returning to study or if it was a viable economic option, taking respite from employment.

A key recovery milestone was reached when women grew in confidence and self-belief, enabling them to recognize and confront people in their lives that they saw as exerting disproportionate influence. In relation to parenting choices and styles, four women spoke of rejecting the preferred methods of others and asserting themselves. A sense of empowerment emerged when they were able to exert control over their baby's wellbeing. For one woman, ensuring that her own mother was not verbally or physically violent toward her in her infant son's presence was of paramount importance, and she asserted herself around her expectations through threatening to withdraw access to her son.

Another participant reflected on receiving what she defined as a “one size fits all” (P5) approach to parenting and attributes much of her mental anguish to the circumstances of her adoption. Being able to successfully breast feed her baby for 18 months was an empowering experience for this mother, and perhaps the most powerful example of her defying her adoptive mother with great success for the health and wellbeing of herself and her son.

Living on her in-law's rural property, another woman (P2) explained how she withdrew from her husband's parents as a strategy to maintain her independence and to reduce the feelings of inadequacy her mother-in-law instilled. In declining offers of child care from the children's grandmother, this woman sacrificed the potential for respite from her two young children, in the interests of sustaining her need for independence and autonomy.

Self-expression through choosing unusual clothing was how another participant asserted her own style. Creativity was employed to physical represent her mood, and she shared how her curious outfits at times draw smiles from community members, which then made her feel “that little bit happier.” On one occasion she related dressing in a pink ball gown with a purple top hat to go to the supermarket:

“I just got up feeling… I'm in a mood today, what can I wear? And I will spend hours because I need to find (the right outfit) and I won't wear something that won't match my personality, if I don't I think that matches my personality for today l won't wear it” (P7).

### Speaking Out

The category of speaking out included disclosing, becoming a mental health advocate/educator, addressing stigma and challenging stereotypes. Although in speaking out, mental illness became dominant in women's personal identity, it was viewed positively and enabled social identity through peer networks to flourish. Enduring the ups and downs of mental illness was seen as a valuable asset that enabled women to take on an educator role, to connect with others through shared experience and to be knowledgeable in ways that others were not.

“My mental health journey has meant that I've got experience in that to be able to connect with people on that level and that's what I want to be able to do” (P13).

Sometimes the desire to engage in community education stemmed from experiences of discrimination that woman believed arose from ignorance.

“I was angry with the way society treated us. And just cast us aside like we were nothing… you can't necessarily see the pain that we carry with us. Its soldiers. And that's what I call it. We're all soldiers. We're all in this together…” (P1).

When women began speaking out, they embraced their illness as a core component of self. The illness symptoms, treatments, and ramifications were integrated into their lives and were a part of how they viewed themselves and presented themselves to others:

“…doing the mental illness education was a really big part of my recovery so to go back and give out to the community, our point of view of how we feel, that's recovery too” (P1).

Participants expressed being selective in how and to whom they disclosed. Generally there was a correlation between the relative perceived safety of the participant's social environment and the extent of their disclosing. Women who felt supported and who had a multi-faceted, secure and positive social identity within their community were more likely to fully disclose. Past involvement with child protection was a deterrent that led women to conceal or minimize the impact of their mental illness.

## Discussion

The majority of women who participated in this study described multifaceted identities that represented their mothering and familial roles, their employment or vocational occupations, community relationships, hobbies and interests, religious and cultural status as well as the ways in which they defined their political or social values and attitudes. These descriptions encompass broader dimensions of identity than have previously been reported in studies with this cohort (39). Each of these could be considered positive and socially valued, or negative and socially devalued, with implications for social and personal identity, depending on the perceived competence and autonomy in the role. For instance, self-identifying as either a “good” or “bad” mother. Mothering was found to be a particularly powerful influence on women's self-concept, and participants identified the characteristics of being a “good Mum” as self-sacrifice, being present and enjoying the role. These concepts are congruent with the prevailing Western ideals of mothering that are grounded in traditional gendered roles that are oppressive to women (40). The findings emphasized the importance of identity to recovery, consistent with the CHIME framework (1).

**Figure 1:**
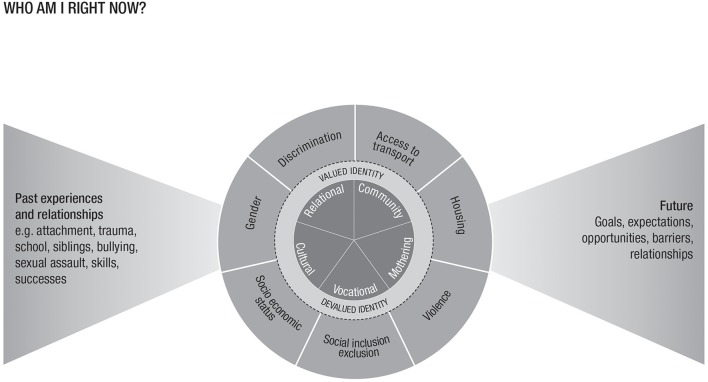
Influences on personal and social identity for mothers with mental illness.

Also significant, were women's expectations of the future. Impending opportunities and obstacles were related to women's ideas about their own competence, social status, agency and aspirations. The findings also highlight that identity is inseparable from social connection with participants described the ways in which their sense of self was heavily shaped by interactions with others (41). Figure 1 represents conceptually the ways in which the women described the factors influencing their personal identity. This figure was developed from the theoretical concepts that emerged from the data. Women described their identity being dependent on how they conceived their self-concept across a range of social domains. Each of these domains could be viewed as socially valued and positive or devalued and negative. Women spoke of being influenced not only by events and relationships from their past, but also their beliefs and aspirations regarding the future, thereby hope was a critical ingredient for a positive identity.

The categories of “doing it my way” and “speaking out” were indicators of significant integration of the illness experience into a woman's personal and social identity. Although the illness identity was at the forefront in “speaking out,” it was constructed positively, intrinsically linked to meaning in life and empowerment, which are other recovery processes identified by Leamy et al. (1). Women emphasized recovery outcomes attained through being supported to identify their strengths, validate and normalize their challenges and self-reflect on the development of their identity across their life span. Often, however, prejudiced attitudes of others, especially associated with their mothering capacity, undermined women's self-concept.

### Mothering Identity

Consistent with previous parenting studies [e.g., (26)], participants spoke of disconnection from other mothers. This was associated with socio-economics, parenting styles and difficulty infiltrating closed (well-established) social groups. Feeling disconnected, socially isolated and “different” is a common experience for mothers with mental illness [e.g., (42)] and is considered a barrier to both sustaining a positive social identity and to recovery (43) and wellbeing more generally (41).

Identifying as a “good” mother was symbolic of women's reflective capacity and internalization of social and cultural expectations as they related to the morality of parenting and the quality of relationships to children. Narratives reflected how women compared their mothering experiences to archetypes represented in their social environment. This finding echoed Venkataraman and Ackerson's (44) study on sources of parenting norms in popular culture, the media and parenting literature. Importantly, the signals women perceived from service providers, portrayed a deep understanding of how society assesses “good” mothering, and an intense desire to be viewed as competent. This facet of personal identity was susceptible to women's own internalized self-criticisms, in addition to the censorious messages conveyed by health professions or family members.

Having a psychiatric diagnosis was a core component of identity for some women, however this was not always experienced negatively, as disclosure could also bring meaning, purpose and connection, when used to educate, support or advocate around mental illness. For other women, having experienced mental health difficulties was just one relatively insignificant facet of a rich and varied life history. In this study a distinction between positive and negative identity was apparent, however this was not necessarily associated with the extent to which women embraced the illness as part of their personal identity.

### Identity, Mental Illness and Trauma

The data also parallels Agnew and colleagues' study that “highlighted the complex and intertwined nature of traumatic experience, personality organization, and self/identity” [(19), p. 8]. Crucially important in defining a self-concept within the current study, were women's experiences from the past, including trauma resulting from physical, psychological or sexual abuse, the quality of early life attachments, transience in housing and schooling, relationships with siblings and parents, experiences at school and access to physical and economic resources. These factors, along with the ways in which women conceptualize them have also been previously highlighted (32, 42, 45, 46). In this study, these factors shaped the identity journey and the way the women saw themselves in the present.

For some women, having a diagnosis provided meaning and understanding that made sense of their symptomatic experiences. Additionally, this enabled them to connect with a peer network of others who shared similar thoughts, feelings and behavior and this offered validation. Mental health education and activism constituted a positive interpretation of lived experience that could simultaneously maintain the mental illness part of identity at the forefront.

### Identity and Change

Identity is assumed to be fluid and dynamic within self-categorization theory (7), and while the participant interviews in this study are a snapshot in time, identity was described by participants as flexible, changing and evolving. This was evident in women's descriptions of themselves over time, and the ways in which they connected with others including disconnecting from unhealthy relationships and becoming more discerning or alternatively, learning to trust. However, there was also reference to consistent and enduring components of identity associated with preferences and strategies for managing stress and mental health difficulties. Crossley (47) adopted a narrative approach in investigating the disruptive impact of trauma, identifying the capacity for trauma to unseat previously coherent conceptualizations associated with self. Crossley (47) found that assumptions regarding one's usual patterns of thoughts, behavior and emotions are undermined along with one's temporal awareness that ordinarily provides meaning and context. This is congruent with women's descriptions of managing distress, in the early period of the illness. For the participants in Crossley's study, narratives become prominent in creating meaning, when customary psychological processes fail under the vast strain of traumatic occurrence.

Not only was it observed that events from women's past shaped their current identity, but their perceptions of their future lives including hopes, plans and aspirations were also influential in defining how they perceived themselves in the present. Identity development appeared to be a non-linear process that was highly permeable to social influence.

#### Recommendations for Research, Policy and Practice

The findings demonstrate mothering identity to be important for mental illness treatment and recovery. Women primarily related as mothers in articulating their self-concept and strove to be recognized as proficient in this role, highlighting their skills, strengths and underpinning parenting values as critical components of their identity. Therapeutic interventions need to explore mothering relationship to self and others, as “therapeutic understanding that takes into account the deficiencies within diagnostic criteria and acknowledges the diverse nature of self and identity of an individual may improve the therapeutic relationship” (19). Within such interventions, women need to feel safe and secure to explore their identity “journey” including the interactions and events that have led to their current self-concept.

A strong therapeutic alliance is critical to cultivating a safe space within which women can begin to address the issues that underpin their healing and recovery (48). Women spoke of the importance of this particularly in the early parenting phase, while adjusting to a new mothering role, and not yet feeling confident in their parenting knowledge and skills. The women who participated in this study indicated that validation of normalcy of parenting challenges can be useful at this time, as well as a more conversational approach that moves beyond the assessment checklists, to the development of an authentic relationship that offered individualized support, rather than reinforcing a sense of being “monitored” and judged.

Elevating the significance of identity and self may reveal areas of intervention that can support more flexible, nuanced and realistic expectations surrounding women's multiple roles and activities. Identity work, integrating past experiences that may challenge individual's assumptions regarding their identity (43) and reflecting on emotional and behavioral responses, is acknowledged as a core component of the recovery process (1, 48).

Beyond this, the current research suggests that investigation of identity and self in the context of social and environmental conditions must incorporate critical reflection on the dominant norms that may be oppressive and result in devalued status within various identity domains. Challenging and contextualizing these dominant assumptions may be particularly significant for marginalized population groups (e.g., Indigenous, people identifying as GLBTI), who are demonstrated to be at increased risk of developing mental health difficulties (49).

Discussions at this deeper conceptual level may be perceived as challenging to establish in the midst of psychological distress, and practitioner judgment in ascertaining readiness for such discussions is essential. However, avoiding these topics can pathologise individual responses to issues that are associated with layers of structural inequality. Future research should also focus upon overcoming workforce barriers within mental health and family services to engaging in identity work with women who have a mental illness.

Mental health policy needs to recognize and reflect the importance of identity work as a crucial part of practice. A disproportionate focus on medication and risk management within clinical mental health continues to stifle recovery oriented practice that encompasses a holistic view of people including consideration of their diverse and multi-faceted roles and relationships.

## Conclusion

The importance of developing and sustaining an identity that is multifaceted and socially valued has been under-acknowledged within mental health services, despite positive identity development being repeatedly identified as a key characteristic of the recovery process. Fostering a positive self-concept, particularly associated with one's parenting role, can assist in facilitating personal recovery in mental illness.

For women who are mothers with mental illness, gendered norms around the mothering role can result in the imposition of unrealistic expectations of women's functioning, particularly if they are living in impoverished social and economic circumstances. Identity work needs to incorporate consideration of the personal level including past experiences, relationships, thoughts, emotions and behavior, as well as the broader environmental context.

## Ethics Statement

This study was carried out in accordance with the recommendations of the Australian National Statement on Ethical Conduct in Human Research. The protocol was approved by South West Healthcare Multi-disciplinary HREC, Monash University HREC and Ballarat Health Services and St John of God Hospital HREC. All subjects gave written informed consent in accordance with the Declaration of Helsinki.

## Author Contributions

RH, DM, and MG developed the research design and methodology together. RH undertook the literature review and the data collection and led data analysis. DM and MG contributed to data analysis and all researchers were involved in initial coding and conceptual analysis as well as developing the findings. RH wrote the manuscript with editing/contributions from DM and MG.

### Conflict of Interest Statement

The authors declare that the research was conducted in the absence of any commercial or financial relationships that could be construed as a potential conflict of interest.
