# Supplementary material for: Identity in Personal Recovery for Mothers With a Mental Illness
Source: Front Psychiatry. 2019 Mar 8;10:89. doi: 10.3389/fpsyt.2019.00089 (PMC6418025; doi:10.3389/fpsyt.2019.00089)
Supplement: Supplementary file 1 [file Data_Sheet_1.docx]

**Interview Schedule**

Could you give me a little bit of background about your mental health difficulties?

Did you have any mental health concerns or problems before you became a mother?

Tell me about your childhood…

What does recovery mean to you?

What do you think might be the key ingredients/steps/processes for personal recovery from mental illness?

How would you describe yourself to someone who maybe doesn’t know you?

Have you always been like that?

What sort of things are important to your sense of self across your lifespan?

Do you think how you see yourself and your sense of self is important for your recovery?

How would (a significant person in your life) describe you?

Who are the people that influence the way you see yourself and who are the people that are really important to you?

What are the important things to you in life?

How are you “different”?

How does your cultural identity influence how you see yourself? Is it about culture, language?

What does being a mum mean to you?

What sort of mother are you?

How did your main roles change once you had a baby?

What was it like, becoming a mother?

Has becoming a mother changed the way you see yourself?
